# P43 Improving therapeutic attainment with continuous vancomycin infusions in critical care: a quality improvement project

**DOI:** 10.1093/jacamr/dlag102.049

**Published:** 2026-06-26

**Authors:** Anna Tilley, Jemima Osborne, Susan Flynn, Fiona Lynch

**Affiliations:** Manchester University NHS Foundation Trust, Manchester, UK; Manchester University NHS Foundation Trust, Manchester, UK; Manchester University NHS Foundation Trust, Manchester, UK; Manchester University NHS Foundation Trust, Manchester, UK

## Abstract

**Background:**

Vancomycin is a time-dependent antimicrobial commonly used in critically ill patients. Continuous infusion strategies optimize time above the MIC and reduce nephrotoxicity. Manchester University NHS Foundation Trust (MFT) guidance targets plasma concentrations of 15–25 mg/L. A baseline audit was completed to ascertain attainment of therapeutic concentrations which showed a high prevalence of sub-therapeutic levels. This prompted a guideline update to have include more dose banding and increase loading and maintenance doses and. A re-audit was completed to assess if there was improved attainment of therapeutic plasma concentrations.

**Methods:**

A retrospective, trust-wide re-audit was conducted across adult critical care units. The baseline audit was completed for prescriptions between September 2022 - September 2024. The re-audit was for prescriptions between May - November 2025. Patients were only included if they had a plasma concentration recorded. Exclusion criteria were age under 18 years, absence of a documented loading dose, patients on intermittent dialysis. Data collected included patient demographics, weight, renal function, loading dose, maintenance infusion rate, timing of plasma concentration measurement, plasma concentration result.

**Results:**

A total of 131 patients were included in the baseline audit, and 58 patients in the re-audit. In the baseline audit, 44% of patients achieved a therapeutic plasma concentration on first measurement, with 53% sub-therapeutic and 2.3% supra-therapeutic. In contrast, following guideline update in 2025, the re-audit showed 55% of patients achieved therapeutic concentrations on first measurement, with sub-therapeutic levels reduced to 17%. However, 29.3% of patients had a supra-therapeutic level. Correct loading doses were prescribed in 87% of cases in the baseline audit and 95% of cases in the re-audit. Correct maintenance infusion rates were prescribed in 88% in the baseline audit but only 71% in the re-audit. In the re-audit, there were 11 supra-therapeutic levels where the guideline was correctly followed. 7 of these (64%) levels were mildly above the target range with levels between 25.1 and 27.5. Importantly, review of supratherapeutic cases did not demonstrate evidence of vancomycin associated nephrotoxicity. Renal function subgroup analysis showed no consistent pattern predicting sub- or supra-therapeutic concentrations when prescriptions were correct. Analysis of the re-audit identified several prescribing and administration errors contributing to supra-therapeutic levels, including incorrect maintenance rates based on renal function and discrepancies between prescribed and administered infusion rates. Overall, the guideline update was associated with a marked reduction in sub-therapeutic exposure, albeit with an increase in supra-therapeutic levels.

**Conclusions:**

Updating the continuous vancomycin infusion guideline significantly reduced sub-therapeutic exposure in critically ill patients. However, due to increased complexity in the new guideline, prescribing and administration errors are a key contributor to supra-therapeutic levels. Targeted education for prescribers and nursing staff is required to improve compliance to the guideline. There is also potential for further guideline refinement, to optimize safe and effective vancomycin use.

